# Identification of the external branch of the superior laryngeal nerve during minimally invasive video-assisted thyroidectomy

**DOI:** 10.1016/S1808-8694(15)31330-6

**Published:** 2015-10-20

**Authors:** Rogério A. Dedivitis, André V. Guimarães

**Affiliations:** ^1^ Ph.D. in Medicine, Post-graduation in Otorhinolaryngology and Head and Neck Surgery, UNIFESP – Escola Paulista de Medicina; Faculty Professor, Discipline of Otorhinolaryngology and Head and Neck Surgery, Universidade Metropolitana de Santos, Santos; ^2^ Master, Post-graduation in Health Sciences, Hospital Heliópolis, Sao Paulo, Professor of the Discipline of Otorhinolaryngology and Head and Neck Surgery, Universidade Metropolitana de Santos, Santos

**Keywords:** thyroidectomy, minimally invasive, video-assisted, superior laryngeal nerve

## Abstract

**T**he minimally invasive video-assisted thyroidectomy (MIVAT) without gas infusion is considered safe and has advantages in terms of cosmetic results compared to the conventional approach. **Aim**: to present our findings regarding the identification of the external branch of the superior laryngeal nerve (EBSLN) during MIVAT. **Study design**: Transversal cohort study. **Material and Method**: twelve patients underwent hemithyroidectomy for thyroid nodular disease through MIVAT method. The upper pedicle of the thyroid was dissected under the magnified view at 0-degree five-millimeter endoscope in order to achieve the identification of EBSLN in all cases. **Results**: We identified 10 (83.3%) EBSLN out of 12 cases. The nerve ran medially to the branches of the superior thyroid artery in 8 cases (80%) and crossed anteriorly in 2 (20%). **Conclusions**: We identified the EBSLN in 83.3% of the cases, whose course was medial to the branches of the superior thyroid artery in 80% and crossing anteriorly in 20%. The ligation of the upper pedicle of the thyroid can be performed under direct view of the EBSLN.

## INTRODUCTION

Since the first report of video-assisted endoscopic subtotal parathyroidectomy in 1996^1^, a technique for the thyroid approach has been investigated^2^. It consists on the creation of a space using minimally invasive methods in which pre-thyroid muscles are pushed aside laterally with a retractor and there is no gas insufflation. Minimally invasive video-assisted thyroidectomy (MIVAT) is considered a safe procedure with esthetical advantages compared to the conventional procedure^3^.

The external branch of the superior laryngeal nerve (EBSLN) innervates the cricothyroid muscle, which is the tensor of the vocal folds. Its damage may lead to reduction in fundamental frequency reach and vocal fatigue after excessive vocal use^4^. In conventional thyroidectomy, the visualization of the branch is not made as a routine owing to its small diameter (0.2 millimeters) and variable course; however, in MIVAT the magnified vision through the optic lens allows its identification^5^.

The purpose of the present study was to present the findings of the identification of EBSLN during MIVAT.

## MATERIAL AND METHOD

From June to August 2001, 12 consecutive patients were submitted to hemithyroidectomy by MIVAT owing to thyroid nodule disease at the Service of Head and Neck Surgery, Hospital Ana Costa, Santos, as provided by the following criteria^6^: thyroid nodule of up to 35 millimeters in its longest axis, absence of ultrasound and lab signals of thyroiditis and maximum total thyroid volume of 20 milliliters, measured by ultrasound.

### MIVAT surgical technique

We based on the technique recommended by Miccoli et al.^7^, with some modifications. We did not require hyperextension of the neck with a pad. After subcutaneous infiltration with bupivacaine solution at 0.5% with adrenalin for anesthetic blockage and vasoconstriction 1:100,000, we made a 2cm transversal incision 2-3cm above the sternal furculum. Using wide dissection and separating pre-thyroid muscles with retractors, we got to the thyroid isthmus. We then used a 5ml endoscope, zero degree, Storz® and spatula and debrider-aspirator for the dissection between the thyroid capsule and the muscle. We dissected the upper thyroid vascular pedicle aiming at identifying the external branch of the superior laryngeal nerve. The pedicle was connected using a harmonic knife UltraCision and clamps Ethicon® CS14C. The recurrent laryngeal nerve was then identified and preserved and the hemithyroidectomy was concluded. None of the patients required the use of drains.

## RESULTS

**Figure 1:**
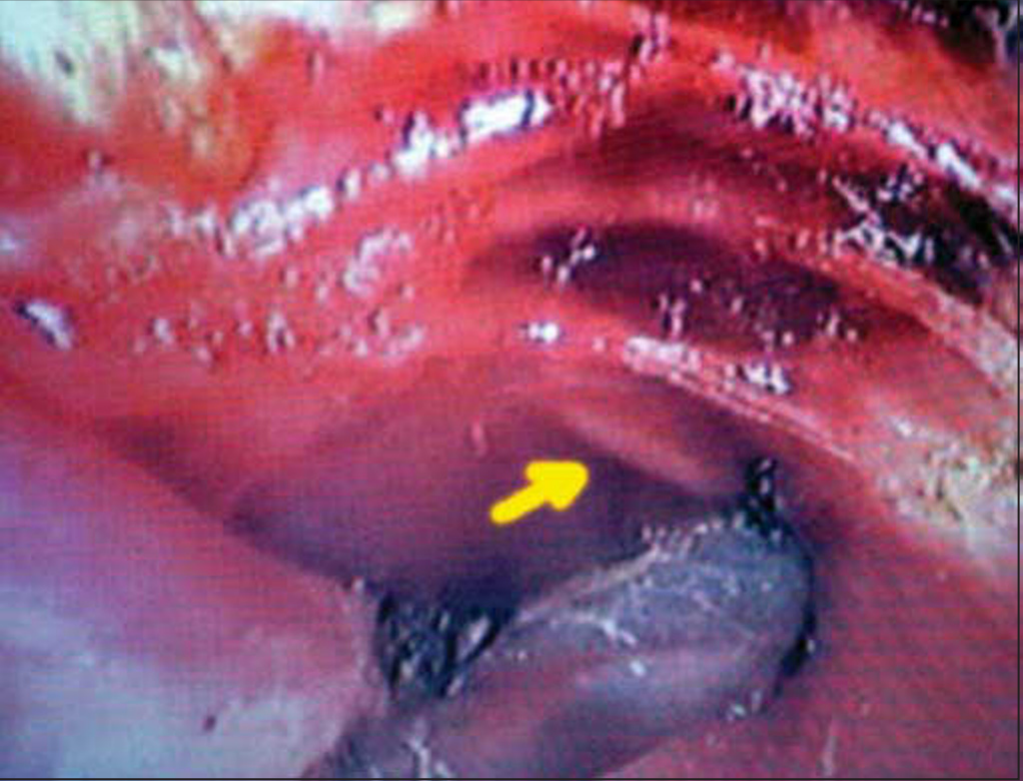
Visualization of the external branch of superior laryngeal nerve medially to the superior thyroid pedicle.

Out of 12 cases submitted to hemithyroidectomy, we identified EBSLN in 10 cases (83.3%), and its path was medial to the superior thyroid artery branches in 8 cases (80%) (Figure 1) and crossed anteriorly such branches in 2 cases (20%). In the 10 studied cases, ligation of thyroid superior pedicle was made using an ultrasonic knife while EBSLN was under direct view.

## DISCUSSION

The median duration of the hemithyroidectomies was 55 minutes. The use of the harmonic scalpel in all cases was considered safe and represents a factor that saves time in the thyroid surgery^8^. The advantages of MIVAT are to reduce neck scar that remains after conventional thyroidectomy and to improve anesthesia, and as a consequence, postoperative recovery^6^. Pain relief is explained by the smaller area of dissection, characteristic of minimally invasive procedures, as well as no head hyperextension. The esthetical advantage is obviously given: the final size of the scar is reduced by half. As to cost, we should point out that the use of video material and disposable harmonic scalpel with clamps tend to increase the cost of the video-assisted procedure. Conversely, the reduction in surgical time and use of anesthetics in groups that have already mastered the technique, is a source of saving. The impact on length of stay is questionable, because in both procedures it tends to be quite short.

Similarly to conventional thyroidectomy, in MIVAT we perform systematic identification, dissection and preservation of recurrent laryngeal nerve. Moreover, it is possible to visualize the external branch of the superior laryngeal nerve as a routine in video-assisted procedures; it is identified without active search in 65% of the cases^5^.

It allows ligation of superior pedicle when the branch is under direct view, with the support of harmonic scalpel. In conventional thyroidectomy, the surgeon tries to make the ligation of the superior pedicle as close as possible to the superior pole of the gland, because the nerve normally runs about 1 cm above the pole region. Previous reports showed that 14 to 20% of the nerves cross the vessels below the superior pole of the gland, which is even more frequent in very voluminous cases^9^. Since all our cases had volumes below 20ml and nodules smaller than 3.5cm in the longest axis, it explains the fact that we did not identify the branches below the level of the superior thyroid pole.

## CONCLUSION

We identified EBSLN in 83.3% of the cases, and the path was normally medial to the branches of the superior thyroid artery in 80% of the cases and crossed anteriorly such branches in 20% of them. Ligation of the superior thyroid pedicle was made with direct view of EBSLN.
